# CRISPR/Cas9 mediated Y-chromosome elimination affects human cells transcriptome

**DOI:** 10.1186/s13578-024-01198-5

**Published:** 2024-01-30

**Authors:** Ludovica Celli, Patrizia Gasparini, Ginevra Biino, Laura Zannini, Miriana Cardano

**Affiliations:** 1 Istituto di Genetica Molecolare “Luigi Luca Cavalli-Sforza”, CNR, 27100 Pavia, Italy; 2 Epigenomic and Biomarkers of Solid Tumors, Department of Research, Fondazione IRCCS Istituto Nazionale dei Tumori, Via Venezian 1, 20133 Milan, Italy; 3 Present Address: Institute for Biomedical Technologies, National Research Council, Via Fratelli Cervi 93, 20054 Segrate, Italy

**Keywords:** Y chromosome, Sex differences, RNA-sequencing, Transcriptome, CRISPR/Cas9

## Abstract

**Background:**

Sexual dimorphism represents a key concept in the comprehension of molecular processes guiding several sex-specific physiological and pathological mechanisms. It has been reported that genes involved in many disorders show a sex-dependent expression pattern. Moreover, the loss of Y chromosome (LOY), found to be a physiological age-driven phenomenon, has been linked to many neurodegenerative and autoimmune disorders, and to an increased cancer risk. These findings drove us towards the consideration that LOY may cause the de-regulation of disease specific networks, involving genes located in both autosomal and sex chromosomes.

**Results:**

Exploiting the CRISPR/Cas9 and RNA-sequencing technologies, we generated a Y-deficient human cell line that has been investigated for its gene expression profile. Our results showed that LOY can influence the transcriptome displaying relevant enriched biological processes, such as cell migration regulation, angiogenesis and immune response. Interestingly, the ovarian follicle development pathway was found enriched, supporting the female-mimicking profile of male Y-depleted cells.

**Conclusion:**

This study, besides proposing a novel approach to investigate sex-biased physiological and pathological conditions, highlights new roles for the Y chromosome in the sexual dimorphism characterizing human health and diseases. Moreover, this analysis paves the way for the research of new therapeutic approaches for sex dimorphic and LOY-related diseases.

**Supplementary Information:**

The online version contains supplementary material available at 10.1186/s13578-024-01198-5.

## Background

Several epidemiologic studies show that sex plays an important role in disease incidence, onset age, progression and therapeutic responses. Males and females differ at the chromosomal level for the presence of XY sex determination system, in which females bear two X chromosomes and males have one X and a Y chromosome. The presence or absence of Y chromosome, epigenetic modifications and the unequal dosage of X chromosome partly affect the sexual dimorphism present in health and disease, as well as sex hormone contribution [1, 2]. It has been established that the Y chromosome, considered a genetic wasteland so far, plays a role in determining these differences in most common pathological conditions such as cancer, Alzheimer, Parkinson and cardiovascular diseases [3]. In particular, the sexual dimorphism in cancer incidence and survival, where males show a higher risk and a lower survival than females, has been attributed to circulating hormones, sex chromosomes, as well as to differences in DNA damage response pathways [4, 5]. Moreover, mosaic loss of Y chromosome (LOY) has been observed in several aging-related conditions, such as Alzheimer disease and cancer, in which LOY increases the susceptibility and risk to develop these diseases [6–10]. Sex chromosome biases have not been considered from the majority of experimental studies since a long time, excluding sex and the presence of the Y chromosome in males as biological variables [11]. In fact, the presence of the Y chromosome could affect the expression of genes located on X and autosomal chromosomes involved in pathways that are associated with specific diseases, and, if dysregulated, coordinate the onset and the progression of a particular condition. In fact, deregulated expression of autosomal genes, including those involved in cell cycle progression, apoptosis, kinetochore functions and mitosis, and DNA damage response expression, has been previously associated with LOY [12, 13]. Thus, our aim was to create a syngeneic human cellular model depleted of the Y chromosome using the CRISPR/Cas9 technology [14], to further deepen the male chromosome role and the consequences of its loss in health and disease. CRISPR/Cas system is a defence mechanism occurring in bacteria and archea. Specifically, type II bacterial CRISPR/Cas9 system has been engineered into a tool consisting of the Cas9 nuclease and a single guide RNA (sgRNA) that targets Cas9 to genomic regions to induce double-stranded DNA breaks, which in turn are repaired by nonhomologous end-joining or homology-directed repair [15]. Due to its simplicity, easy modification, and high efficiency, CRISPR/Cas has derived several reliable genome editing tools, not only for cancer screening, diagnosis, and clinic treatment, but also applicable to basic research [16]. Since previous studies demonstrated the efficacy of CRISPR/Cas9 method to induce multiple DNA double strand breaks (DSBs) to delete an entire chromosome, we decided to apply this technique for the creation of a Y-depleted cell line [14, 17]. Additionally, we performed RNA-sequencing (RNA-seq) on the Y-deficient cell line, for tracing a specific gene expression pattern, when compared to the original wild type (WT) cell line. This transcriptome analysis is crucial to understand the role of Y chromosome in the sexual dimorphism characterizing human health and many common diseases. Moreover, beyond the sex related studies, the targeted Y chromosome elimination technique can be seen also as a novel strategy to treat human aneuploidy diseases.

## Results

### Targeted elimination of human Y chromosome

To investigate the role of the human Y chromosome in both healthy tissues and diseases, we decided to generate a syngeneic non-transformed human cell model depleted of the male specific chromosome, from the male retinal epithelial ARPE-19 cell line. Since it was originated from a normal human retinal pigmented epithelium (RPE) bearing a single Y chromosome, never immortalized and quite rapidly growing, this derived cell line is suitable for a mostly unbiased analysis of the differential transcriptome [18, 19].

**Fig. 1:**
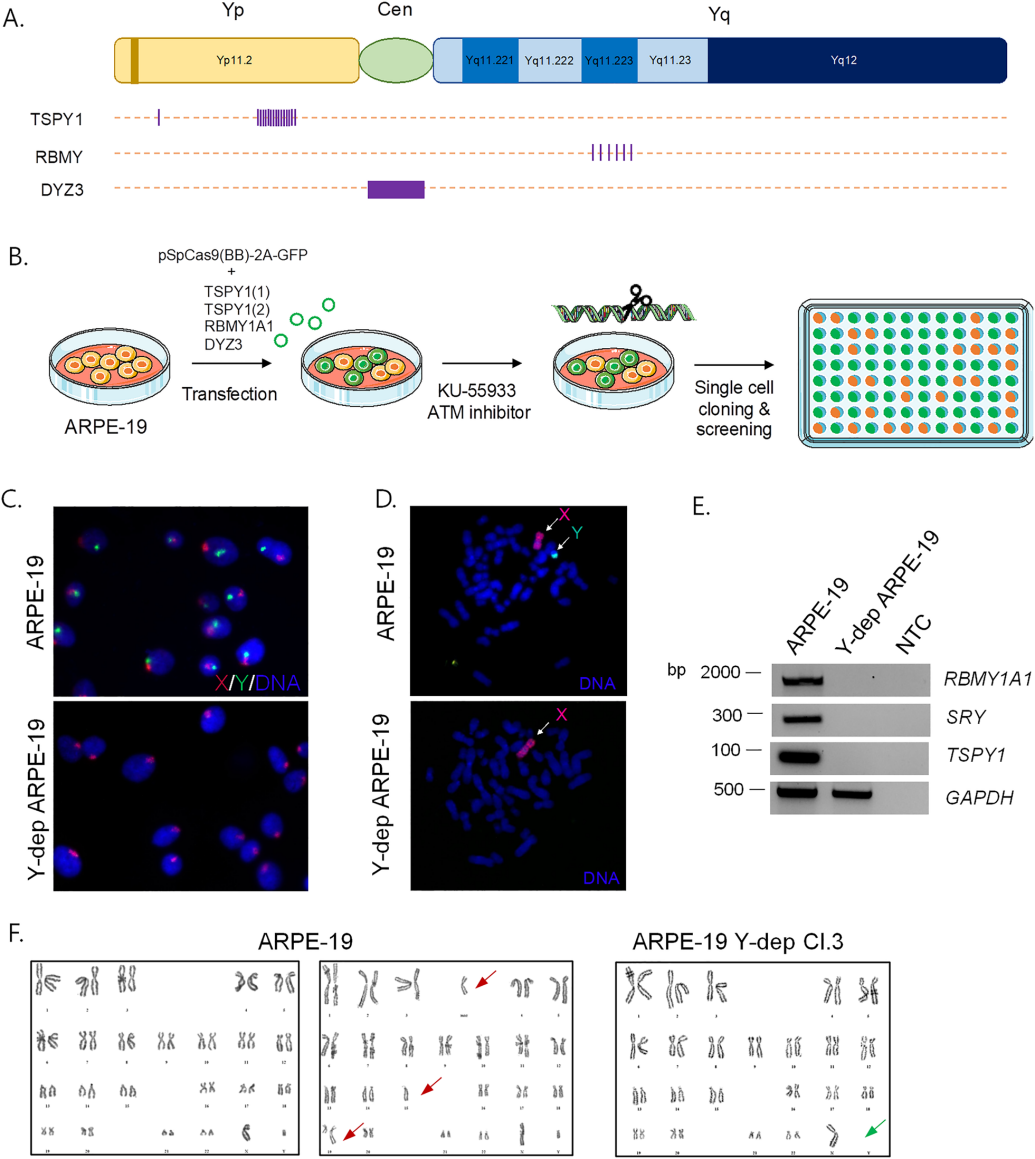
Targeted elimination of human Y chromosome. **A** gRNAs targeted sites on human Y chromosome are indicated. **B** Experimental plan: a mixture of pSpCas9(BB)-2A-GFP vectors containing gRNA sequence for *TSPY1*, *RBMY1A1* and *DYZ3* was used to transfect ARPE-19. Cells were treated for 16 h after transfection with the ATM inhibitor KU-55933. After subcloning, single clones have been picked, expanded, and then screened by FISH. The Figure was partly generated using Servier Medical Art, provided by Servier, licensed under a Creative Commons Attribution 3.0 unported license. **C** DNA-FISH analysis on interphase and metaphase (**D**) spreads indicating the complete loss of Y chromosome. Green whole chromosome paint (WCP) probe was used for Y chromosome hybridization. Red WCP probe was used for X chromosome identification. **E** Agarose gel electrophoresis of *RBMY1A1*, *SRY* and *TSPY1* PCR products derived from ARPE-19 Y-negative clone 3. GAPDH has been used as reference control gene. **F** Karyotype analyses of ARPE-19 and Y-negative clone 3. Red arrows indicate chromosomal alterations in ARPE-19 cells. Green arrow shows Y chromosome elimination in Y-depleted clone 3

Previous studies demonstrated the efficacy of CRISPR/Cas9 method to induce multiple DSBs as a new approach to delete an entire chromosome [14, 17]. Therefore, we searched for tandemly repeated sequences across the human Y chromosome and we decided to target testis specific protein Y-linked 1 (*TSPY1*), that is repeated ~ 35 times in the short arm (Yp11.2), RNA binding motif protein Y-linked family 1 member A1 (*RBMY1A1*), that is repeated 6 times in the long arm (Yq11.223), and the DYZ3 locus located at the centromere with 24 cuts [20] (Fig. 1A). The necessary gRNAs (two sequences for TSPY1, one for RBMY1A1 and one for DYZ3) have been designed at chopchop.cbu.uib.no [21] and have been inserted in the pSpCas9(BB)-2A-GFP plasmid [22]. A mixture of the generated vectors was used to transfect the non-transformed ARPE-19 cells and, to efficiently prevent the repair of the induced DSBs, we treated them for 16 h after transfection with the ATM inhibitor KU-55933 [14] (Fig. 1B). After subcloning, single clones have been picked, expanded, and then screened by fluorescence in situ hybridization (FISH) technique to detect the Y chromosome elimination, using a whole-chromosome probe (green). FISH analyses demonstrated the complete loss of Y chromosome in both interphase and metaphase spreads, and the complete absence of any Y chromosome translocation to other chromosomes in three different analysed clones, hereafter defined as clones 3, 5, and 9 (Fig. 1C, D; Additional file 1: Fig. S1A). In addition, the effective elimination of Y-linked genes, such as SRY, TSPY1 and RBMY1A1, in these clones was further confirmed by end-point PCR on genomic DNA (Fig. 1E). We also karyotyped ARPE-19 cells and the three different Y-depleted clones. We found that, as already reported in the literature [23], parental ARPE-19 metaphase spreads are heterogeneous since they show both normal and rearranged karyotypes (Fig. 1F). Differently, in the Y-depleted ARPE-19 clone 3 we observed the majority of cells with 45, X0 chromosomes and some cells with 44, X0 karyotype, caused by the loss of a random chromosome (Fig. 1F). In clones 5 and 9, the karyotype analysis revealed the majority of cells with 45, X0 chromosomes and some cells with 40–44, X0, therefore displaying a random loss of 1–5 chromosomes (Additional file 1: Fig. S1B). Hence, these results demonstrate that we established near-isogenic ARPE-19 cellular systems in which the most significant variable is the absence of the Y chromosome. Importantly, these cellular models display different degrees of chromosomal instability.

### The absence of the Y chromosome affects the transcriptome

**Fig. 2:**
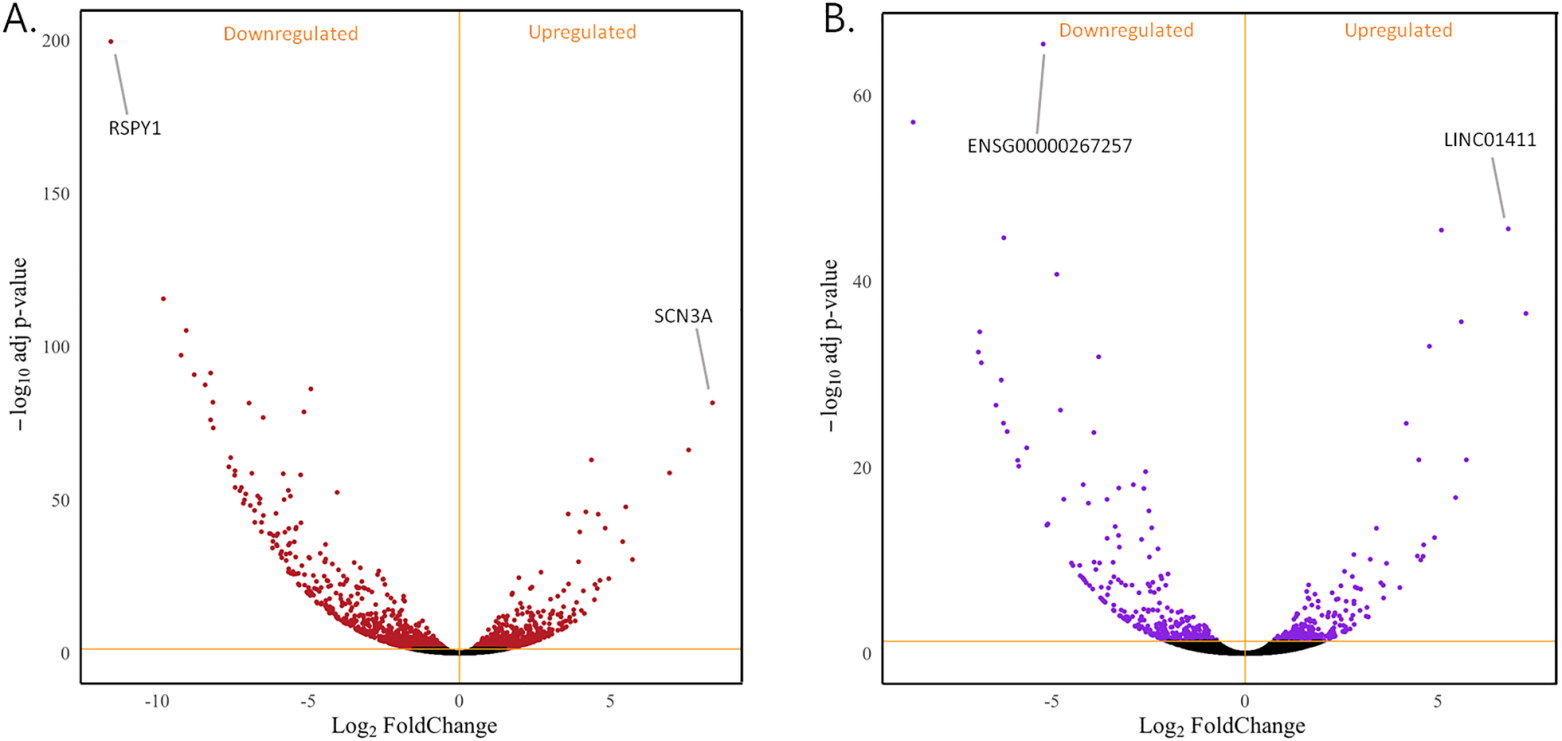
Differential transcriptome analysis between ARPE-19 WT and ARPE-19 Y-depleted cell lines. Volcano plots representing regulated genes in the two conditions. Significantly differentially expressed genes (P ≤ 0.05 of Benjamini–Hochberg correction of nominal P-values obtained from the applied two-sided Wald test). Top up- and down-regulated genes are indicated in both volcanos. **A** Representation of DE PCGs. Significantly differentially expressed genes are shown in red. **B** Representation of DE lncRNAs. Significantly differentially expressed genes are shown in purple

To understand if and how the Y chromosome influences the expression of other sex-linked and autosomal genes, possibly impacting on pathways associated with specific diseases, we analysed the transcriptome of the WT and engineered ARPE-19 clone 3 cell lines. To this aim, we extracted RNA from both the WT and the Y-deficient cells and assessed the gene expression profile of the samples through RNA-sequencing. Comparative analysis of the Y-deficient versus the WT cell line identified significant (according to Benjamini Hochberg corrected P < 0.05) differential expression of 2384 genes in total, of which 1854 were protein coding genes (PCGs) and 530 were long non-coding transcripts (Additional file 2: Table S1; Fig. 2A, B). As expected, we found no expression of Y-linked genes in the Y depleted ARPE-19 clone 3, with the most downregulated gene being the Ribosomal Protein S4 Y-Linked 1 *(RPS4Y1),* whose haploinsufficiency plays a role in the Y negative Turner Syndrome [24]. The high expression of *RPS4Y1* in the WT ARPE-19 cells makes the gene the most suitable internal control for the actual absence of Y genes in the Y-depleted ARPE-19 clone 3. In addition, we also found altered expression, not only of genes localized on the X chromosome, but also on autosomes. The top-most upregulated PCG is sodium voltage-gated channel alpha subunit 3 *(SCN3A),* located on chromosome 2, while long intergenic non-protein coding RNA 1411 *(LINC01411)* and *ENSG00000267257* are the top up- and down-regulated lncRNAs, located on chromosome 5 and 18, respectively. The Principal Component Analysis (PCA) on gene expression tables shows that the samples separate according to the condition on the first principal component (PC1), that explains the 78% and 70% of the variance in PCGs and lncRNAs, respectively (Additional file 1: Fig. S2).

Collectively, these results are consistent with the karyotype of our cell line and indicate that the lack of Y chromosome can influence the transcriptome of non-transformed human cells.

### Transcriptome analysis reveals sex-biased regulated pathways

**Fig. 3:**
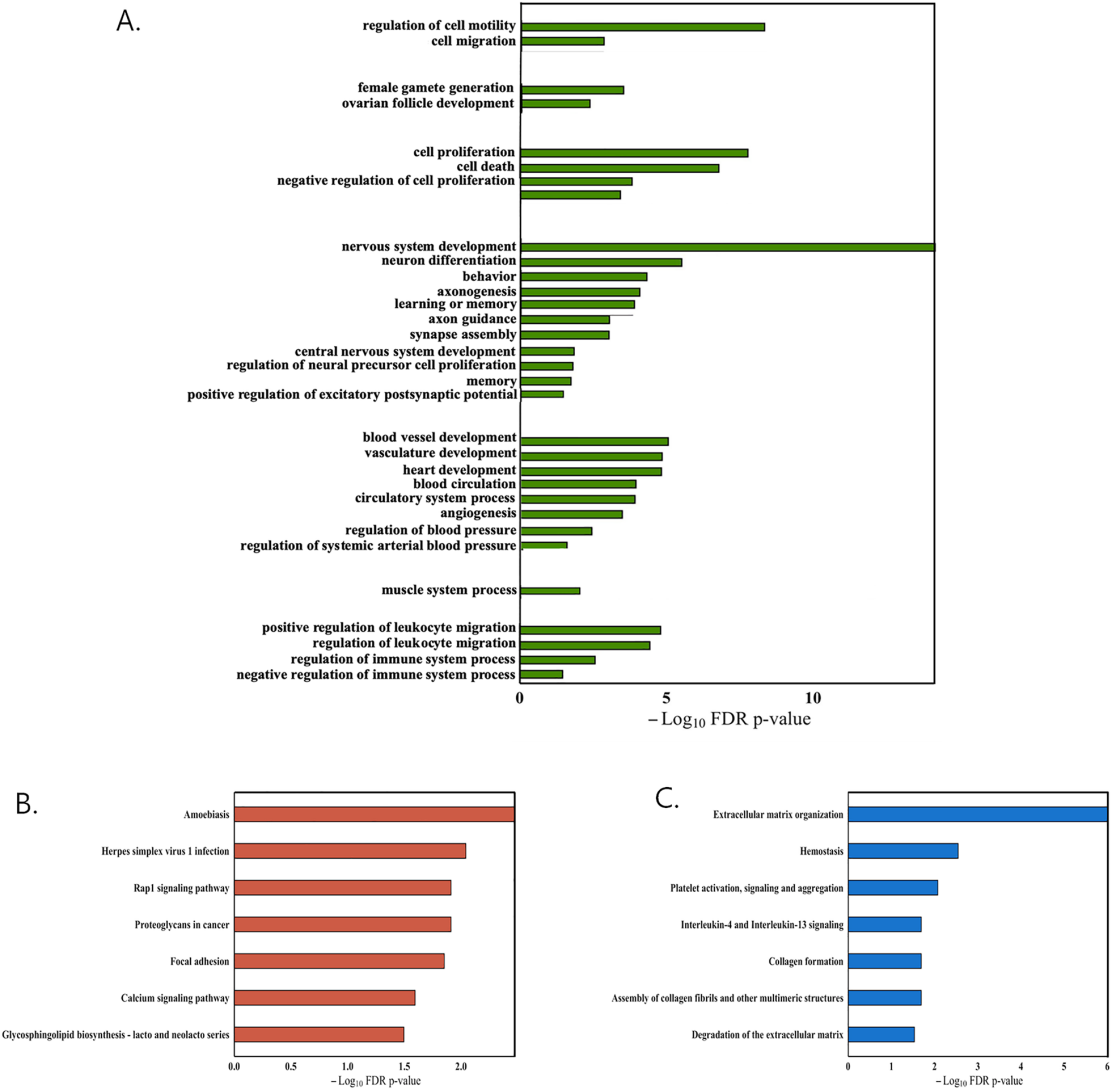
Functional enrichment analysis of differentially expressed PCGs between ARPE-19 WT and ARPE-19 Y-depleted cell lines. **A** Plot showing the GO-BP terms enriched in the DEG list (FDR values are depicted in the –log_10_ scale on the X axis). **B** Plot depicting the KEGG terms enriched in the DEG list (FDR values are depicted in the –log_10_ scale on the X axis). **C** Barplot reporting the enriched REACTOME pathways (FDR values are depicted in the –log_10_ scale on the X axis)

Protein-coding differentially expressed genes (DEGs) were used to get insights on the biological process and pathways affected by Y chromosome absence. Gene Ontology Biological Process (GO-BP) analysis identified 335 enriched biological processes in the intersection between PANTHER and DAVID annotation tools. Redundant GO-BP terms were pooled together (Fig. 3A; Additional file 2: Table S2). The same differentially expressed PCGs were used to assess their implication in complex pathways in KEGG and REACTOME repositories (Fig. 3B, C; Additional file 2: Table S3). Regulation of cell migration and proliferation resulted as the most represented GO-BP categories (Fig. 3A). Rap1 signalling pathway detected with the KEGG pathway annotation seems to support GO-BP annotation, as it has been demonstrated to be involved in cellular migration [25] (Fig. 3B; Additional file 2: Table S3). Also angiogenesis, blood vessels and heart development GO-BP resulted as enriched processes and, accordingly, cardiovascular diseases are known to be the leading cause of death among women worldwide [26]. In addition, REACTOME pathways concerning the cardiovascular system were significantly enriched, such as “hemostasis” and “platelet activation, signalling and aggregation” (Fig. 3C; Additional file 2: Table S3). These annotations, together, point toward the regulation of processes related to angiogenesis. Interestingly, nervous system development as well as muscle system arose in the GO-BP analysis, consistently with the previously reported expression differences reported in brain and muscle tissues, respectively [27]. The regulation of immune system processes arising in the GO-BP analysis is exacerbated by the presence of terms associated with inflammation in the KEGG pathway annotation. As a matter of fact, terms such as “amoebiasis” and “herpes simplex virus 1 infection” are confirmed in the REACTOME database with the term “Interleukine-4 and Interleukine-13 signaling”. These observations are consistent with the previously reported differences in immune response between males and females [28]. Taken together, these data, united with the ovarian follicle development GO-BP term (Fig. 3A; Additional file 2: Table S2), suggest the female-mimicking nature of the Y-depleted cell line system.

### LncRNAs analysis discloses their involvement in sex-biased pathways and diseases

**Fig. 4:**
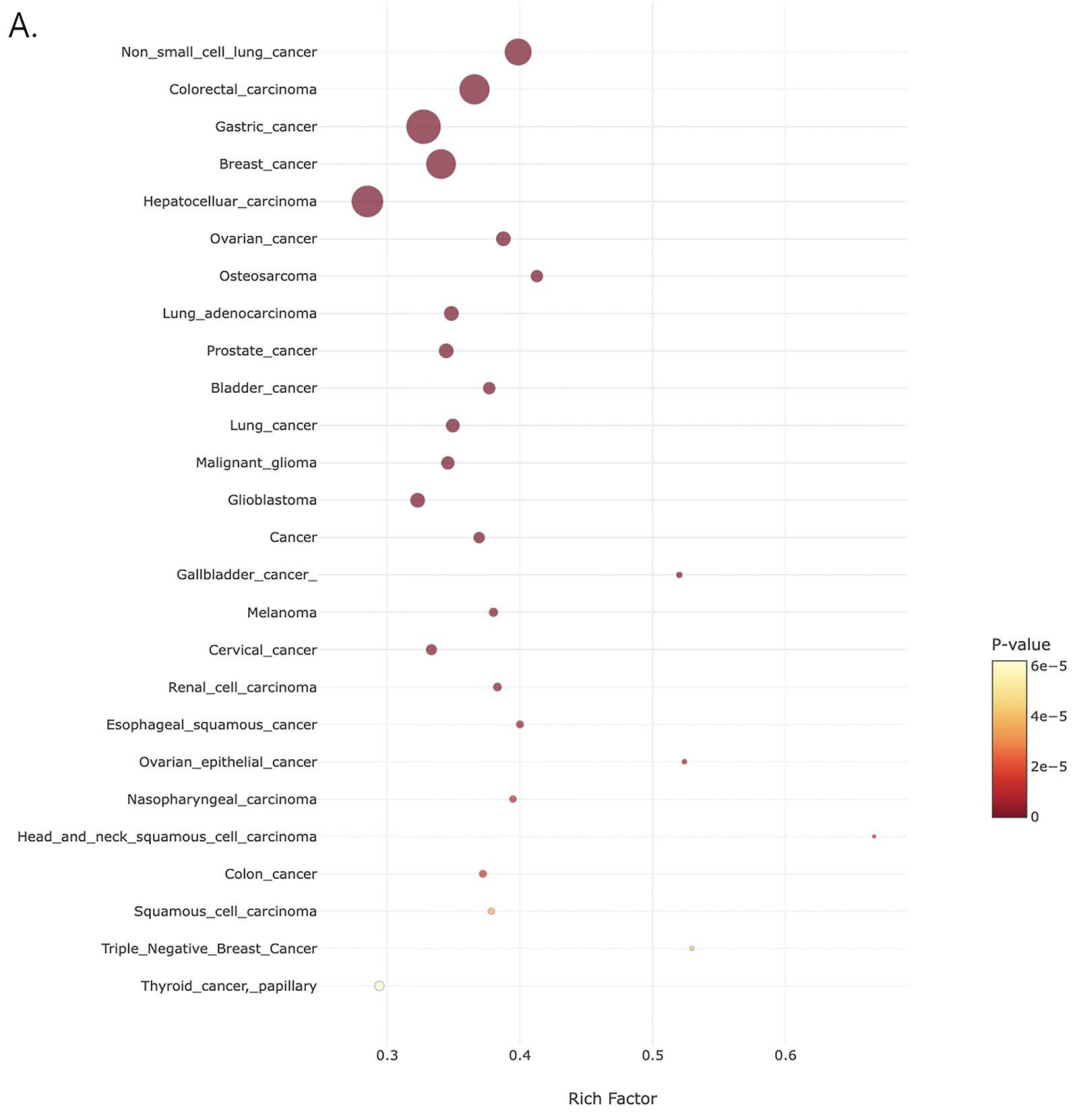
Disease enrichment in DE lncRNA with expansion using TLSEA. **A** Bubble plot representation of the disease enrichment analysis. Significantly enriched disease pathways (P-value ≤ 0.05) are represented as bubbles and the statistical significance of each term is indicated by the color shading in the legend. On the X-axis, the enrichment value (rich factor) is represented. Bubble areas directly correlate with the number of DE lncRNAs and similar belonging to each disease pathway

To understand to which extent the DE lncRNAs are involved in the sex-biased pathways detected through the functional annotation of PCGs, the ncFANs-NET module embedded in the FANs tool was used to generate coding non-coding co-expression networks (CNC) based on all the expression data available in the TCGA and GTEx portal [29]. First, 530 DE lncRNAs and 1854 DE PCGs resulting from the differential expression analysis (DEA) were used. The retained correlations were those showing a Rho > 0.3. This analysis allowed the robust identification of a total of 59 lncRNAs, whose expression is modulated with 712 PCG, giving rise to 3457 co-expression interactions (Additional file 2: Table S4). To enable the investigation of the 7 informative GO-BP classes resulting from the functional annotation (Fig. 3A), the CNC network table obtained was merged with the functional annotation analysis (group 1: cell motility and related terms; group 2: female gamete formation and related terms; group 3: cell proliferation and related terms; group 4: nervous system development and related terms; group 5: blood vessel development and related terms; group 6: muscle system; group 7: immune system and related terms). This analysis revealed that 52 DE lncRNAs are in CNC with PCGs belonging to group 1 and 55 in group 3 (Additional file 2: Tables S5, S7). Consistently with the bond between proliferation and migration mechanisms, 56 lncRNAs were in common between the two classes. Among them, for example, EPB41L4A divergent transcript *(EPB41L4A-DT)* overexpression has been linked with depression in migration of hepatocellular carcinoma and breast, lung and renal cancers [30]. Moreover, urothelial cancer associated 1 *(UCA1)* and PRKCQ antisense RNA 1 *(PRKCQ-AS1),* were associated with proliferation, migration and apoptosis, especially in cancers [31, 32]. LncRNAs belonging to group 2 (female gamete formation and related terms) were 16 (Additional file 2: Table S6), of which the testis-specific transcript, Y-linked 14 *(TTTY14),* downregulated in the Y-depleted cell line and the DPPA2 Upstream Binding RNA *(DUBR)* reported to be overexpressed in testis and ovary, respectively [33, 34]. Group 4 reports 58 DE lncRNAs (Additional file 2: Table S8), of which *FOXD2-AS1* has been associated with resistance to chemotherapeutic treatments in gliomas [35], TIPARP antisense 1 *(TIPARP-AS1)* was demonstrated to regulate AhR signaling, which in turn is involved in neuroendocrine pathways such as those that control the brain-pituitary-interrenal and gonadal axes [36, 37] and long intergenic non-protein coding RNA 1615 *(LINC01615),* biomarker of head and neck squamous cell carcinoma [38]. Interestingly, group 5 (blood vessel development and related terms), consisting of 55 DE lncRNAs (Additional file 2: Table S9), contains the above mentioned *TIPARP-AS1* and *FOXD2-AS1.* Indeed, *FOXD2-AS1* has shown an involvement in the progression of hemangioma [39], while the regulation of AhR signaling is related to the control of blood pressure and *TIPARP-AS1* modulates it [36, 37]. They also share the ST3GAL6 Antisense RNA 1 *(ST3GAL6-AS1),* whose dysfunctions are associated with myelomas, consistently with the blood circulation related terms [40]. Thirty-nine DE lncRNAs are coexpressed with genes involved in muscle system process (group 6; Additional file 2: Table S10), with MAGI2 antisense RNA 3 *(MAGI2-AS3)* reported as associated with the diaphragmatic region [41]. A total of 54 DE lncRNAs were found in group 7 (Additional file 2: Table S11), with the TGFB2 antisense RNA 1 *(TGFB2-AS1)* regulating the signaling via *TGF-β,* a well-known regulatory cytokine [42]. Since lncRNAs are being investigated for their role in the pathogenesis of diseases, their known involvement in them have been investigated through the TLSEA software. Among the most enriched diseases, based on the similarity matrix (coefficient of similarity = 0.9), we found breast cancers, gliomas and thyroid cancers as well as ovarian, among the others (Fig. 4A; Additional file 2: Table S12). Despite the lack of annotation and the general predictive nature of the results coming from lncRNAs, these data overall suggest their contribution to the regulation of the sex-biased pathways emerging from the DEA on ARPE-19 and the isogenic Y-depleted cell line.

### Validation of differentially expressed genes in Y-depleted ARPE-19 clones

**Fig. 5:**
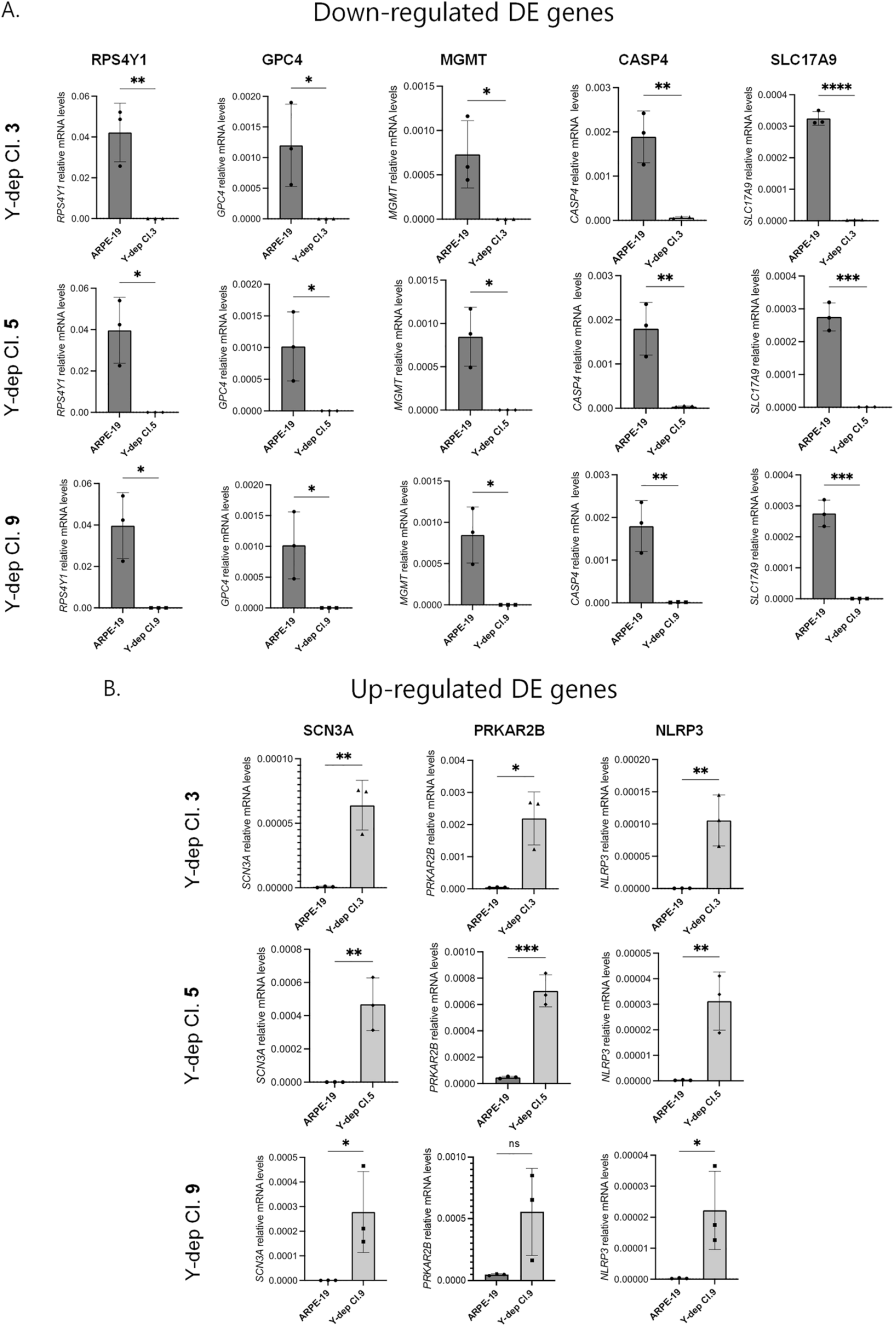
Validation of down- (**A**) and up- (**B**) regulated genes resulted from the RNA-seq analysis. RT-PCR analyses were performed in triplicate by using RNA extracted from ARPE-19 Y-depleted clones 3, 5 and 9 VS ARPE-19 WT. Each bar represents mean ± SD calculated from three independent experiments. P values were derived from Student’s t test between the indicated samples (ns: P > 0.05; *P ≤ 0.05; **P ≤ 0.01; ***P ≤ 0.001; ****P ≤ 0.0001)

To validate our transcriptome analyses, the expression profiles of genes identified in the RNA-seq were analysed by measuring the relative mRNA levels of eight genes, among the top significant ones, in the three Y-depleted isolated clones. mRNA samples derived from Y-depleted ARPE-19 clone 3, clone 5 and clone 9 were compared with WT ARPE-19 (a sample set different from RNA-seq has been used for ARPE-19 and Y-depleted clone 3; Fig. 5). By using quantitative RT-PCR, we confirmed the down-regulated expression of *RPS4Y1*, glypican 4 (*GPC4*), O-6-methylguanine-DNA methyltransferase (*MGMT*), caspase 4 (*CASP4*), solute carrier family 17 member 9 (*SLC17A*9) and the upregulation of *SCN3A*, protein kinase cAMP-dependent type II regulatory subunit beta (*PRKAR2B*), NLR family pyrin domain containing 3 (*NLRP3*) in ARPE-19 Y-depleted clones 3, 5 and 9 compared to WT ARPE-19. Our results showed that the data from qRT-PCR were consistent with those of RNA-sequencing and were well reproduced in all the different Y-depleted cellular lines.

To evaluate the clinical relevance of our findings and cellular model, we analyzed the expression of the top deregulated genes also in the neuroblastoma cell line SK-N-BE(2) and in its derivative Be(2)-C which lost the Y chromosome. As shown in the charts (Additional file 1: Fig. S3) all the tested genes, except for *CASP4,* resulted deregulated in Y negative cells and four of them (*RPS4Y*, *GPC4*, *MGMT* and *SLC17A9*) demonstrated the same trend observed between ARPE-19 and the Y-depleted clone 3, therefore supporting the relevance of our model.

### Validation of differentially expressed genes in female cells

**Fig. 6:**
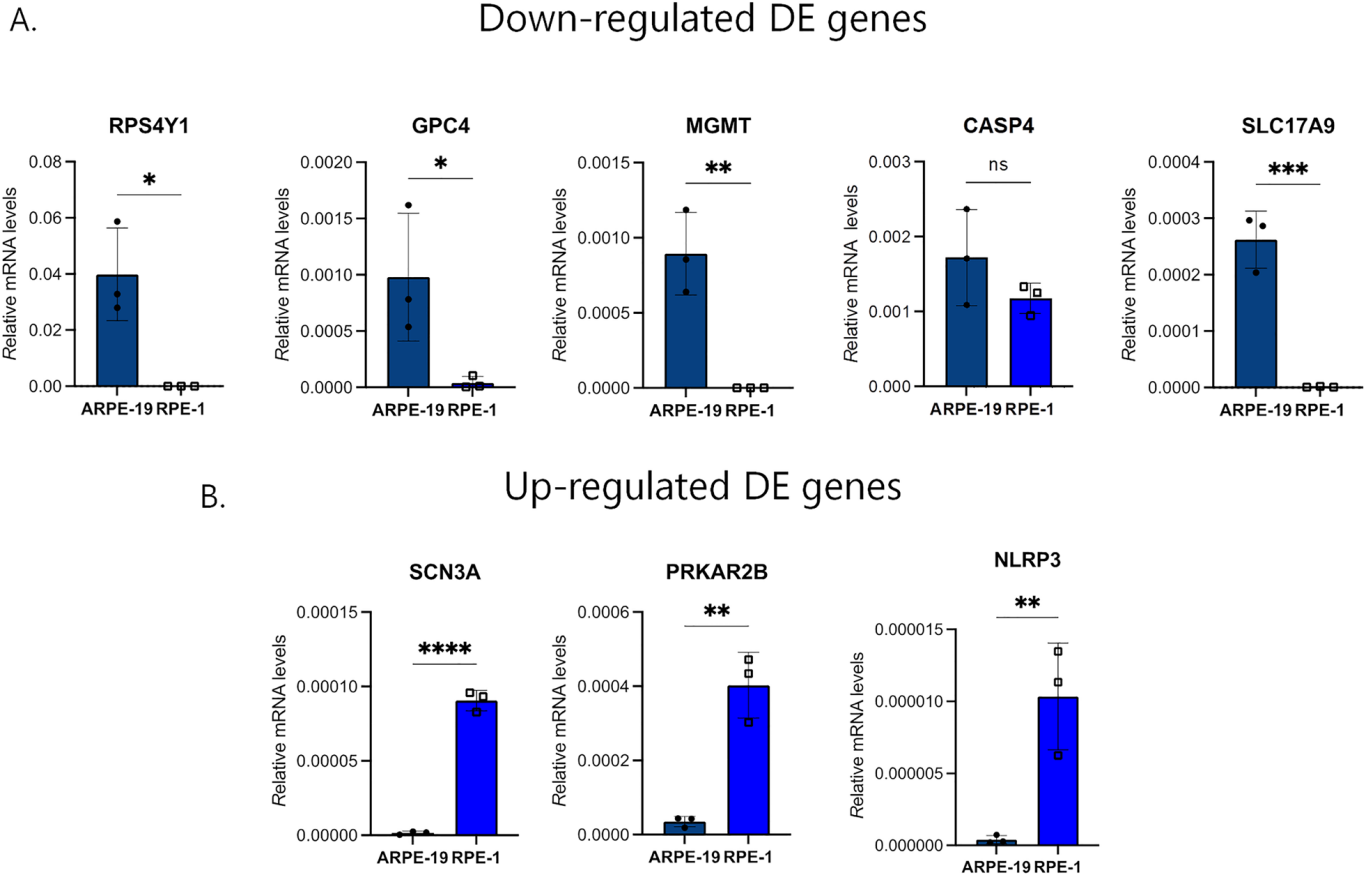
Validation of down- (**A**) and up- (**B**) regulated genes in RPE-1 female cells. RT-PCR analyses were performed in triplicate by using RNA extracted from RPE-1 VS ARPE-19 WT. Each bar represents mean ± SD calculated from three independent experiments. P values were derived from Student’s t test between the indicated samples (ns: P > 0.05; *P ≤ 0.05; **P ≤ 0.01; ***P ≤ 0.001; ****P ≤ 0.0001)

There is strong evidence from the DEA and the GO-BP analysis demonstrating the female-mimicking profile of the Y-depleted generated system. Thus, to further strengthen our results and investigate the female-like nature of our Y-negative cell line, we validated the transcriptome analyses using female cell line RPE-1, which shares the same histotype of ARPE-19. To this purpose, we measured the relative mRNA levels of the same previously analysed eight top significant genes in RPE-1 cells compared to ARPE-19 (Fig. 6). The quantitative RT-PCR data showed that the transcript levels of RPE-1 genes significantly follow the same trend of Y-depleted clone 3 mRNA levels compared to WT ARPE-19, except for *CASP4* levels, possibly due to cell type variability. These results suggest a female-like behaviour of X0 ARPE-19 cells and further support the Y chromosome dependency of the differential regulation of the transcriptome observed.

### Y chromosome depletion promotes cell migration and invasion and reduces cell growth

**Fig. 7:**
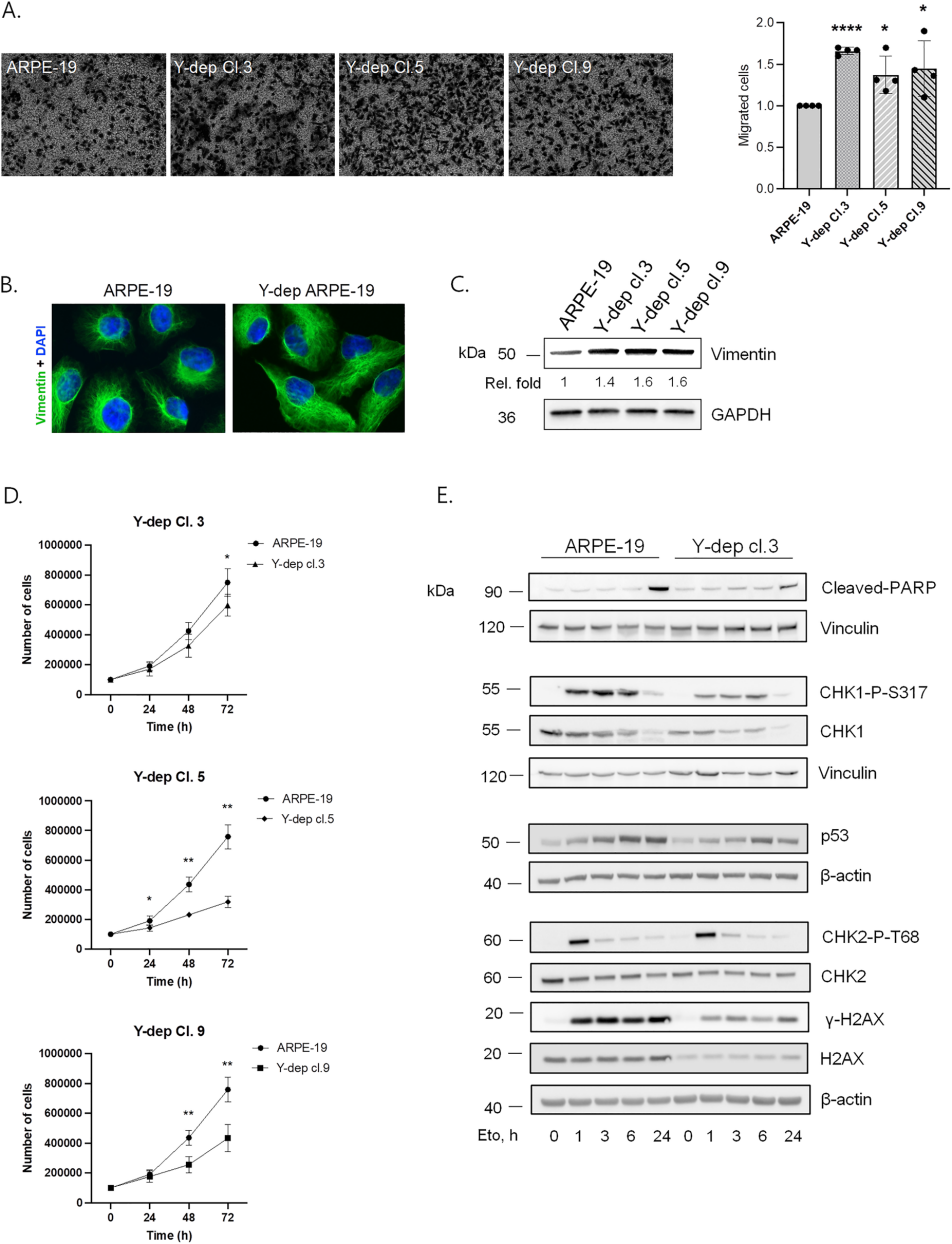
Y chromosome depletion promotes cell migration, reduces cell growth and affects the DDR. **A** Transwell migration assay followed by crystal violet staining in ARPE-19 and Y-negative clones. Cell migration has been quantified by elution of crystal violet staining with 0,1% SDS and measured at 570 nm absorbance. Each bar represents mean ± SD calculated from four independent experiments. P values were derived from Student’s t test between the indicated samples (ns: P > 0.05; *P ≤ 0.05; **P ≤ 0.01; ***P ≤ 0.001; ****P ≤ 0.0001). **B** Representative images of immunofluorescence analysis of vimentin signal (green) in ARPE-19 and Y-depleted clone 3. **C** Representative images of western blot analysis of vimentin protein levels in ARPE-19 cells and Y-negative clone 3, 5 and 9. **D** Cell proliferation curves in ARPE-19 cells compared to Y-negative clones 3, 5, and 9. Cell counts were carried out at 24, 48 and 72 h after seeding. Means ± SD calculated from five independent experiments are shown. Statistical analysis was performed using Mann–Whitney non-parametric test (*P ≤ 0.05; **P ≤ 0.01). **E** Representative images of western blot analyses of DDR factors in ARPE-19 cells and Y-depleted clone 3 after 1, 3, 6 and 24 h of etoposide treatment

GO-BP enrichment analysis identified cell migration regulation as one of the most represented pathways. Thus, we hypothesize that WT and Y-negative ARPE-19 cells could exhibit different ability to migrate, which is also a marker of epithelial mesenchymal transition (EMT). To verify this, we performed Transwell cell migration assays on X0 and XY cell lines and the obtained data were quantified through crystal violet staining and measured at 570 nm (Fig. 7A). As shown in the plot, the migratory ability of Y-depleted cells was significantly enhanced and well reproduced in all X0 cellular clones. These data were confirmed by immunofluorescence and western blot analyses showing higher vimentin levels in Y-depleted ARPE-19 compared to WT cell line (Fig. 7B, C), which correlate with enhanced cell migration and EMT [43]. Further, we evaluated the invasion capacity of Y-depleted clone 3 through matrigel invasion chambers, revealing an increased number of invading cells compared to XY cell line (Additional file 1: Fig. S4). Overall, these results indicate that LOY induces a higher cell migratory ability and invasion potential, possibly implicated in malignant transformation and metastasis. In addition, during our studies we observed that cells that lost the Y chromosome were duplicating slower than the XY cell line. This proliferative impairment was also suggested by the transcription analysis, by the upregulation of Cyclin Dependent Kinase Inhibitor 1A (*CDKN1A,* better known as p21) gene, which mediates cell cycle arrest, and the downregulation of Extracellular matrix protein 1 (*ECM1*), which has been found to promote cell proliferation [44]. To experimentally verify this observation, we measured the ability of ARPE-19 cells and Y-negative clones to proliferate. To this aim, ARPE-19 and X0 clones 3, 5 and 9 were seeded in duplicate and counted 24, 48 and 72 h later. As expected, the results demonstrated a slower growth rate in all the Y-depleted cell clones compared to WT ARPE-19 (Fig. 7D).

### Y chromosome depletion affects DNA damage response to etoposide treatment

Sexual dimorphism has been recently linked also to differences in DNA damage response (DDR) pathway, a complex signalling cascade responsible for detecting DNA lesions and triggering the appropriate molecular response, thus preventing tumor initiation and progression [4]. Moreover, some DDR-related genes have been found to associate with LOY [12]. To further characterize the role of Y chromosome in this crucial signalling pathway, we carried out a time course western blot analysis of DDR-related proteins in ARPE-19 WT and Y-depleted clone 3 after 1, 3, 6 and 24 h of etoposide treatment, a genotoxic agent inducing DSBs [45]. The results showed that LOY globally reduces the levels of DDR proteins such as the tumor suppressor p53, the apoptotic marker cleaved-PARP, the Chk1 and Chk2 kinases, and histone H2AX (Fig. 7E; Additional file 1: Fig. S5). We also assessed the levels of phosphorylated Chk1 on serine 317 and Chk2 on threonine 68, representing activated proteins participating in the DDR phosphorylation cascade, and levels of histone H2AX phosphorylation at serine 139 (γ-H2AX), a sensitive marker of DNA damage. Even if the phospho-proteins levels in Y-depleted cells seem to be decreased too, when normalized on their respective total protein, they appear to be induced, as reported in the densitometric bar graphs (Fig. 7E; Additional file 1: Fig. S5), suggesting an intricate regulation of such modifications in Y negative cells.

## Discussion

Experimental studies have been male-centric for years, often excluding sex and the Y chromosome as biological variables from research, treatments and clinical trials. Despite this, the Y chromosome's role has been recently recognized to contribute to sex differences in health and disease. Here, we engineered the ARPE-19 cell line with CRISPR/Cas9 system to specifically destroy the Y chromosome targeting specific repeated regions, to study the gene expression pattern when compared with the WT cell line. However, it is important to consider that also the epigenetic variability or sex-specific targeting by transcription factors could contribute to increase the sex differences between men and women [2], in differentially expressed genes or not. The Y chromosome elimination and the absence of Y-related genes, previously detected through FISH and PCR analyses, were further confirmed by RNA-sequencing, with the system resembling the X0 phenotype, as it is demonstrated by Y-linked genes downregulation, especially the *RSP4Y1.* The functional annotation analysis on the DEGs between the XY and the Y-depleted cell line highlighted peculiar sex-biased pathways, that may be however specific for ARPE-19 cells. In particular, terms related to cardiovascular, muscle, nervous and immune system appeared as the most enriched. For example, physiological differences in the skeletal muscles between males and females are a certainty and include energy metabolism, fiber type composition, and contractile speed [46]. Male muscles have a higher capacity for anaerobic metabolism united with higher maximum power output than female muscles. On the other hand, female muscles have been found to be more fatigue resistant and to recover faster than male muscles. Even though these differences appear to be a consequence of the different hormonal background, a mechanistic explanation of their functioning is still missing. Additionally, the disparity observed in the physiology of the two sexes, has an impact also in the pathology of some other systems. Indeed, cardiovascular disease (CVD) has always shown different outcomes according to sex. From data spanning from 1980 to 2010, men have shown increased risk of developing CVD at a younger age and with worse prognosis than women [47]. However, in the last decades, a worsening in the women's outcome has been registered, with CVD becoming the leading cause of death among women worldwide. This stagnation seems to be due to emerging non-traditional risk factors unique to—or more common in—women, like autoimmune disorders and depression among the others [26]. As a matter of fact, 78% of people affected by autoimmune disorders, spanning from systemic lupus erythematosus (SLE) to Hashimoto’s thyroiditis, are women [48–50]. The actual causes underneath this bias are yet to be clearly understood, as it is not confined to sex hormones. The same tendency towards females has been reported also in mental health diseases, such as depression [51]. Even though this inclination has been linked to hormone fluctuations, there are also differences in the expression of genes in the brain tissues between males and females that could exacerbate the tendency towards depression experienced by women [27, 52, 53]. Additionally, despite the overall predictive nature of the results on lncRNAs, they were found to be modulated in the comparison between the engineered cell line and the ARPE-19. Through the analysis of the CNCs and of diseases, we identified a total number of 58 lncRNAs potentially involved in the pathways discussed. Among them, some were already studied as being involved in proliferation, migration, nervous system development, cardiovascular system as well as being implicated in inflammatory diseases and sex-biased cancers or exclusively expressed in sexual organs, while others’ functions are yet to be discovered. Thus, although some results may be specific for this cellular system, our Y-depleted cell line could help in the experimental discovery of lncRNAs functions. The system developed in this work could be also seen as a useful strategy to study Y aneuploidy in tumorigenesis and a potential treatment against a broad spectrum of aggressive cancers. In fact, although LOY has been reported as a para-physiologic aging consequence in elderly men [54], it has also been observed in cancer tissues, where it is associated with an overall worse prognosis. It is the case, among the others, of kidney renal papillary cell carcinoma, esophageal adenocarcinoma and male breast cancer, enumerated amongst the most aggressive malignancies, with high genomic instability, aneuploidy, as well as an augmented mutation burden [55, 56]. LOY has been found to affect also 33–36% of cases of pancreatic cancer and 23–34% of bladder cancer. In the latter case, its higher aggressiveness has been linked to the loss of *KDM5D* and *UTY* genes, displaying tumor suppressive roles [13, 57]. The enhanced migratory and invasive potential that we observed in Y-depleted ARPE-19 is consistent with these observations, as aggressive tumors have shown the ability to colonize different sites. Moreover, the increased cell migration observed in Y-deficient cells is sustained by the downregulation of RAP1 GTPase activating protein 2 (*RAP1GAP2*), whose depletion has been demonstrated to be associated with increased cell migration and invasion [58]. The difference in the migratory ability between the WT and Y-negative cell lines was also supported by the downregulation and the upregulation of respectively *CDH13*, encoding for T-cadherin, and *CDH1*, encoding for E-cadherin. In fact, T-cadherin expression has been found to be reduced in many tumors [59, 60], and its overexpression was linked to migratory inhibition function [61]. Although E-cadherin has been considered as an inhibitor of cell migration [62], evidence has emerged for its role in promoting cell migration and tumor progression [63]. The oncogenic property of Y chromosome loss and its association to a poor prognosis are in accordance with our results on DDR protein levels analyses. The decreased levels of cleaved-PARP, p53 and Chk1 and Chk2 kinases in Y-negative cell line delineate a weaker and poorer response to DNA damage, compared to Y-positive cells. Interestingly, the phosphorylation mediated-activation of the two kinases and the DNA damage marker γ-H2AX are induced in Y-depleted ARPE-19, as if the cells try to fill the gap by amplifying the defective signalling cascade. Indeed, these observations make the Y chromosome a pivotal player in tumor suppression, as already suggested [9]. Moreover, due to the rarity of most aggressive tumors, the establishment of cancer-derived cell lines to be used as a model is challenging in some cases, like male breast cancer. This delays the progress of finding suitable and precise treatments. The Y-depleted cell line system proposed here, able to mimic LOY, could open the way towards a better understanding of the correlation between the Y chromosome absence and the worse cancer prognosis. These possibilities are further supported by our findings that genes deregulated in this cellular model are differentially expressed also in Y-positive and -negative neuroblastoma cell lines, even if not all of them showed the same trend observed in Y-negative ARPE-19 cells. However, these differences are not surprising considering the different tissue origin (brain versus retina) and the transcriptome alterations induced by tumoral transformation. Actually, our developed system may be used to simulate specific cancer cell lines, through the insertion of somatic mutations known as drivers for certain tumor types. Moreover, LOY has been linked to age-related macular degeneration in men and it has been demonstrated to have a driver role in the development of uveal melanoma [8, 64], two examples of diseases strictly related to the retinal tissue; these observations, together with the high frequency of LOY identified in the majority of cancer types in men, and together with our gene expression results in Y-positive and negative cancer cell lines, make Y-depleted ARPE-19 system an efficient model for future studies of clinical relevance.

## Conclusions

Overall, the transcriptome analysis of ARPE-19 Y-depleted cells highlighted a sex-biased nature of the enriched pathways, confirmed by literature. Since a clear mechanism of sex dependent occurrence of many diseases is missing, the developed system could be adapted to understand the role of the Y chromosome in the sexual dimorphism characterizing human health and many common pathologies.

## Methods

### CRISPR/Cas9 and sgRNA production

gRNAs were cloned into the pSpCas9(BB)-2A-GFP plasmid (Addgene 48138) [22]. After BbsI digestion of the plasmid, a pair of oligos for each target sequence were phosphorylated, annealed, and ligated to the vector. The correct insertion was verified performing a PCR using U6 primer mapping on vector sequence and reverse primer from each target followed by Sanger sequencing (Eurofins Genomics). Guides and primer sequences are listed in the table below.

**Table:** 

Sequence name	Target sequence	Primer (Top)	Primer (Bottom)
TSPY1(1)	TGAACGGCCAGCAGCTCCTCCGG	CACCGTGAACGGCCAGCAGCTCCTCCGG	AAACCCGGAGGAGCTGCTGGCCGTTCAC
TSPY1(2)	GTATCCGGATTATGAAGTGGAGG	CACCGGTATCCGGATTATGAAGTGGAGG	AAACCCTCCACTTCATAATCCGGATACC
RBMY1A1	CTCGTGTATTACTATATGCGTGG	CACCGCTCGTGTATTACTATATGCGTGG	AAACCCACGCATATAGTAATACACGAGC
DYZ3	ATGATAGGTTGAACTCCCGGAGG	CACCGATGATAGGTTGAACTCCCGGAGG	AAACCCTCCGGGAGTTCAACCTATCATC

### Cell culture

ARPE-19, Y-depleted ARPE-19, RPE-1, SK-N-BE(2) and Be(2)-C cell lines were maintained in MEM (Lonza) supplemented with 10% FBS, 100 U/ml penicillin and 0,1 mg/ml streptomycin. 10 μg/ml Hygromycin B was added to RPE-1 cells. Cells were maintained at 37 °C and 5% CO_2_.

### Cells transfections and treatments

Transfections were carried out using Lipofectamine 2000 (Thermo Fisher Scientific), according to the manufacturers’ instructions. 10 μM ATM inhibitor (KU-55933) was added 4 h after transfection. For DDR experiments, DNA damage was induced by treating cells with 20 μM etoposide (Merck).

### Karyotype analysis

An adequate quantity and quality of metaphases were obtained, using standardized harvesting protocols from cell line cultures. More specifically, cells were arrested in metaphase by incubation with Colcemid (Life Technologies, Carlsbad, CA, 0.02 μg/ml overnight) and subjected to incubation in hypotonic solution (0.075 M KCl, 0.5 mM EDTA and 20 mM Hepes, pH 7.4) for 1 h at 37 °C. Finally, cells were fixed in Carnoy’s fixative (3:1 methanol to acetic acid). Metaphase spreads were obtained by cells dropped onto microscope glass slides, dried at room temperature and counterstained with Giemsa. Using Olympus BX51 Fluorescent microscope, at least 20 metaphases were captured, analysed and karyotyped with MacKtype (PowerGene, Applied Imaging).

### DNA-FISH

Cells were incubated in hypotonic solution for 45 min at 37 °C, fixed in Carnoy’s fixative, and then dropped onto microscope slides. The slides were incubated at 56 °C for 2 h and then pre-treated with SSC 2X buffer followed by Pepsin-HCl at 37 °C. X and Y chromosome probes (XCyting Chromosome Paints, MetaSystems) were hybridized at 72 °C for 2 min and then at 37 °C overnight. The slides were rinsed for 2 min in SSC 0.4X at 72 °C and washed in 0.05% Tween20/SSC2X RT for 30 s. The slides were stained with DAPI-antifade solution and mounted with a coverslip. Images were acquired using Olympus IX71 microscope with CoolSNAP ES camera.

### PCR

PCR analyses were performed with the GoTaq G2 Flexi DNA Polymerase kit (Promega) according to manufacturer’s procedure and using the following conditions: 95 °C for 5 min, 30 cycles of PCR (95 °C for 30 s, 60 °C for 30 s, 72 °C for 30 s) and 72 °C for 10 min. The amplification products were loaded on 2% agarose gels. Primers used were: SRY_for GCATTCATCGTGTGGTCTCG; SRY_rev TTCGCTGCAGAGTACCGAAG; TSPY1_for CATGACCCCAGAGTCTGCAC; TSPY1_rev CCTTCCTGGCTTGGGCATTA; RBMY1A1_for TGGCTTCCCTCACATGAAG; RBMY1A1_rev TTGCTTCTTGCCACAGCAGAAG; GAPDH_for ACCACAGTCCATGCCATCAC; GAPDH_rev TCCACCACCCTGTTGCTGTA.

### RNA-seq

Total RNAs were extracted using the RNeasy Mini Kit (QIAGEN) and treated with DNase (TURBO DNA-free kit, Thermo Fisher Scientific), according to manufacturer’s instructions. Total RNA was sequenced with the Illumina NovaSeq™ 6000 technology to obtain paired-end reads of 150 bp per mate, in triplicate per condition. Raw fastq files were checked to assess their quality using the FastQC tool (https://www.bioinformatics.babraham.ac.uk/projects/fastqc/). No trimming procedure was applied as no adapter nor low quality calls were detected. Sequencing reads were aligned to the human reference genome GRChg38 assembly, using the STAR software [65]. Alignment was performed in splice-junction aware mode using the genome transfer file (gtf) of GENCODE (https://www.gencodegenes.org/human/gencode.v41.basic.annotation.gtf.gz), available at the time of the analyses. Aligned reads were counted through the featureCounts tool [66], using two different gtf for protein coding (PCGs) and long non-coding RNAs (lncRNAs), respectively. The PCGs gtf file was obtained from the original basic annotation using the R software.

### Differential expression analysis

Differentially expressed genes were detected into two separate analyses for PCGs and lncRNAs exploiting the raw counts generated by featureCounts. The analysis was performed with the “DESeq2” R-software [67], Y-depleted versus XY cell line. Genes were considered differentially expressed having adjusted p-value < 0.05. Principal Component Analysis (PCA) was performed to check for the homogeneity among the samples and for the presence of any extreme outlier.

### Functional enrichment analysis

Differentially expressed PCGs were annotated in DAVID (https://david.ncifcrf.gov/) and PANTHER (http://www.pantherdb.org/) software to seek for enrichment in the gene ontology biological processes (GO BP). Only significantly enriched GO terms in common between the two tools were retained. KEGG and REACTOME pathway annotations were performed using the DAVID tool only. Results were considered for discussion applying a statistical significance threshold of 0.05 based on the multiple testing corrected p-values (Benjamini-Hochberg). Plots representing the enriched terms were obtained using the “ggplot2” R-package, version 3.4.0

### Analysis and prediction of lncRNAs functions

The list of differentially expressed lncRNAs and PCGs resulting from the differential expression analysis were used to generate coding-non-coding networks and through the ncFANs software (http://ncfans.gene.ac/) [29]. Specifically the ncFANs-NET embedded module was run to generate the coding non-coding co-expression network (CNC), getting advantage of the RNA-seq data from 54 normal tissues and 33 cancer types from GTEx [68] and the TCGA portal (https://www.genome.gov/Funded-Programs-Projects/Cancer-Genome-Atlas), respectively. Filters were applied to the correlation coefficient (Rho), while none were applied to the Topological Overlap Measure (TOM), as the software defaults. Resulting data were integrated with the annotation data previously obtained from the functional annotation analysis of PCGs in R environment (R Studio suite). In addition, the ncFANs-NET modules generated GO annotations exploiting the CNCs: the annotations generated confirmed our previous results. To get insights on the involvement of the DE lncRNAs in known diseases, an enrichment analysis with expansion was performed using the TLSEA tool (http://www.lirmed.com:5003/tlsea) [69]. The expansion mode enabled the identification of novel lncRNA–lncRNA association networks by merging lncRNA-related information obtained from multiple sources with the different lncRNA-related similarity (coefficient of similarity = 0.09) networks, exploiting graph representation learning method. Plot and enrichment of diseases were generated.

### qRT-PCR

Total RNAs were extracted as described in the RNA-seq section. RNA was quantified using NanoPhotometer P330 (Implen) and 1 μg was retro-transcribed using the SuperScript IV First-Strand Synthesis System (Thermo Fisher Scientific). qPCR reaction was performed in triplicate using QuantiFast SYBR Green PCR Kit (Qiagen) and LightCycler 480 System (Roche). Samples were normalised using GAPDH gene as a reference. Experiments were repeated three times. Primer sequences were: RPS4Y1_for TCTTCCGTCGCAGAGTTTCG; RPS4Y1_rev TGAGGAAGACGATCAGAGGAA; GPC4_for TCCCTCGCAAATTGAAGCTCC; GPC4_rev GCAACCGCTAAGCCTTGAG; MGMT_for TGAATGCCTATTTCCACC; MGMT_rev CTTCCATAACACCTGTCT; CASP4_for AAGTCCTCTGACAGCACATTCTTGG; CASP4_rev GAGGCAGTTGCGGTTGTTGAATATC; SLC17A9_for AGGGGTTTACTTCCCTGCC; SLC17A9_rev GTCAGCAGCGTCCCAAACT; SCN3A_for TGGATGGAAACAGACTGACC; SCN3A_rev GTGTTCATCATCAGCAAAGTCA; PRKAR2B_for TTCGGCGAACTGGCCTTAATG; PRKAR2B_rev ACTTTCAGGCGTTCAGAAAACT; NLRP3_for TGCCCGTCTGGGTGAGA; NLRP3_rev CCGGTGCTCCTTGATGAGA; GAPDH primers were the same used for PCR.

### Transwell migration assay

Transwell chambers (8 µM, Corning) were used to evaluate cell migratory ability. 1 × 10^5^ cells were seeded in the upper chamber in FBS-free medium and the lower chamber was filled with complete medium supplied with 10% FBS. After 5 h, cells in the upper chamber were removed, and migrated cells embedded in the Transwell membrane were fixed and coloured with crystal violet solution (0,2% crystal violet, 2% EtOH, H_2_O) for 10 min. Stained cells were eluted with 1% SDS, rocking for 10 min, and quantified at 570 nm absorbance. Experiments were repeated four times.

### Invasion assay

Corning® BioCoat® Matrigel® Invasion Chambers with 8.0 µm PET Membrane were used to perform invasion assay. Membranes were rehydrated for 2 h in a 5% CO2 incubator. 1 × 10^5^ cells were seeded in the upper chamber in FBS-free medium and the lower chamber was filled with complete medium supplied with 10% FBS. After 24 h, cells in the upper chamber were removed, and invaded cells embedded in the membrane were fixed and coloured with crystal violet solution (0,2% crystal violet, 2% EtOH, H_2_O) for 10 min. Stained cells were eluted with 1% SDS, rocking for 10 min, and quantified at 570 nm absorbance. Experiments were repeated four times.

### Immunofluorescence analysis

ARPE-19 e Y-negative clone 3 cells were fixed with 4% paraformaldehyde, permeabilized with 0.5% Triton X-100 and saturated in 3% BSA. Cells were incubated with mouse anti-vimentin antibody (Sigma-Aldrich Cat# V6630, RRID:AB_477627; diluted 1:40 in 1% BSA), followed by secondary fluorescent Alexa Fluor 488 conjugated antibody incubation (Thermo Fisher Scientific Cat# A-11001, RRID:AB_2534069; diluted 1:400 in 1% BSA). The coverslips were mounted on glass microscope slides with ProLong Gold with DAPI reagent (Thermo Fisher Scientific), to counterstain nuclei. Images were acquired using Olympus IX71 microscope with CoolSNAP ES camera. Experiments were repeated at least two times.

### Western blot analysis

Western blot analysis was performed on total cell extracts using the NuPAGE system (Thermo Fisher Scientific) or the Mini PROTEAN TGX gels and the Trans-Blot Turbo Transfer System (Biorad). The membrane was blocked with 4% milk solution and blotted with primary antibodies. Antibodies used were: vimentin (Sigma-Aldrich Cat# V6630, RRID:AB_477627; diluted 1:400), GAPDH (Sigma-Aldrich Cat# SAB1405848, RRID:AB_10739020; diluted 1:1000), p53-DO7 (Santa Cruz Biotechnology Cat# sc-47698, RRID:AB_628083; 1:500), cleaved PARP (Cell Signaling Technology Cat# 5625, RRID:AB_10699459; 1:1000), phospho-Histone H2A.X (Ser139) (Millipore Cat# 05-636, RRID:AB_309864; 1:1000), H2AX (Millipore Cat# DR1016-100UG, RRID:AB_437862; 1:1000), vinculin (Sigma-Aldrich Cat# V9264, RRID:AB_10603627; 1:1000), Phospho-Chk1 (ser317) (Cell Signaling Technology Cat# 12302, RRID:AB_2783865; 1:1000), CHK1 (Cell Signaling Technology Cat# 2360, RRID:AB_2080320; 1:1000), β-Actin (Sigma-Aldrich Cat# A1978, RRID:AB_476692; 1:4000), Phospho-Chk2 (Thr68) (Cell Signaling Technology Cat# 2661 (also 2661S, 2661P, 2661L, 2661T), RRID:AB_331479; 1:1000), CHK2 (Abcam Cat# ab109413, RRID:AB_10863751; 1:50000). The results were quantified by densitometry using Fiji software. Experiments were repeated at least two times.

### Proliferation assay

ARPE-19 e Y-negative clones 3, 5 and 9 were seeded in duplicates in 6 wells plates, 100.000 cells each well, and counted 24, 48 and 72 h after seeding. Growth curves were determined and reported in the chart. Experiments were repeated five times.

### Supplementary Information

**Additional file 1: Figure S1.** FISH and karyotype analyses of Y-depleted clones 5 and 9. **Figure S2.** Principal Component Analysis (PCA) graph on gene expression data. **Figure S3.** Validation of top deregulated genes in SK-N-BE(2) and Be(2)-C cells. **Figure S4.** Y chromosome depletion promotes cell invasion. **Figure S5.** Y chromosome depletion affects DNA damage response to etoposide treatment at different time points.

**Additional file 2.** Bioinformatic analyses of RNA-seq data.

## Data Availability

The datasets generated and analysed during the current study are available in the NCBI Sequence Read Archive repository (BioProject ID: PRJNA930361), https://dataview.ncbi.nlm.nih.gov/object/PRJNA930361?reviewer=8u2jcj6vjb32saorss85fg4po0. All other relevant data supporting the findings of this study are included in this published article and its supplementary information files. Source data are provided with this paper.
