# Supplementary material for: CRISPR/Cas9 mediated Y-chromosome elimination affects human cells transcriptome
Source: Cell Biosci. 2024 Jan 30;14:15. doi: 10.1186/s13578-024-01198-5 (PMC10829266; doi:10.1186/s13578-024-01198-5)
Supplement: Supplementary file 1 — Additional file 1: Figure S1. FISH and karyotype analyses of Y-depleted clones 5 and 9. Figure S2. Principal Component Analysis (PCA) graph on gene expression data. Figure S3. Validation of top deregulated genes in SK-N-BE(2) and Be(2)-C cells. Figure S4. Y chromosome depletion promotes cell invasion. Figure S5. Y chromosome depletion affects DNA damage response to etoposide treatment at different time points. [file 13578_2024_1198_MOESM1_ESM.pdf]

Figure S1

A

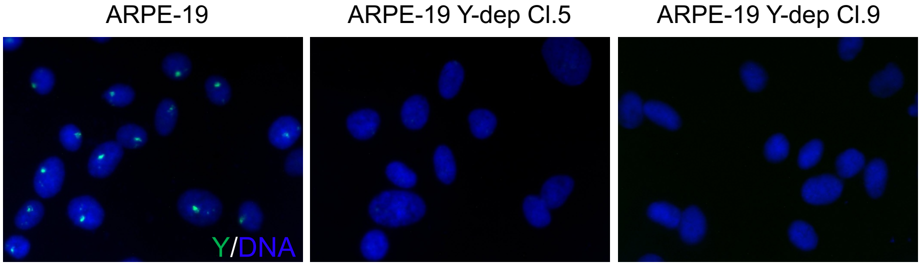

B

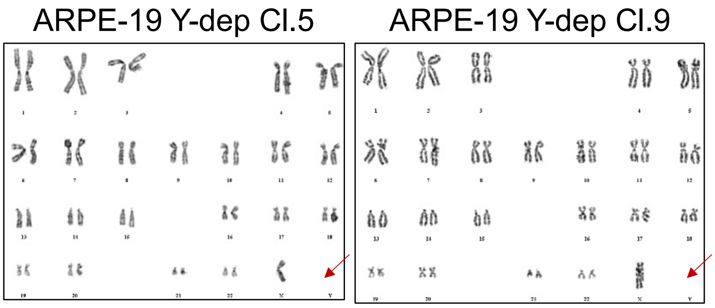

Figure S1. A) DNA-FISH analysis on interphase spreads indicating the complete loss of Y chromosome in Y-depleted ARPE-19 clones 5 and 9. Green whole chromosome paint (WCP) probe was used for Y chromosome hybridization. B) Karyotype analyses of Y-negative ARPE-19 clones 5 and 9. Red arrows indicate Y chromosome elimination.

Figure S2

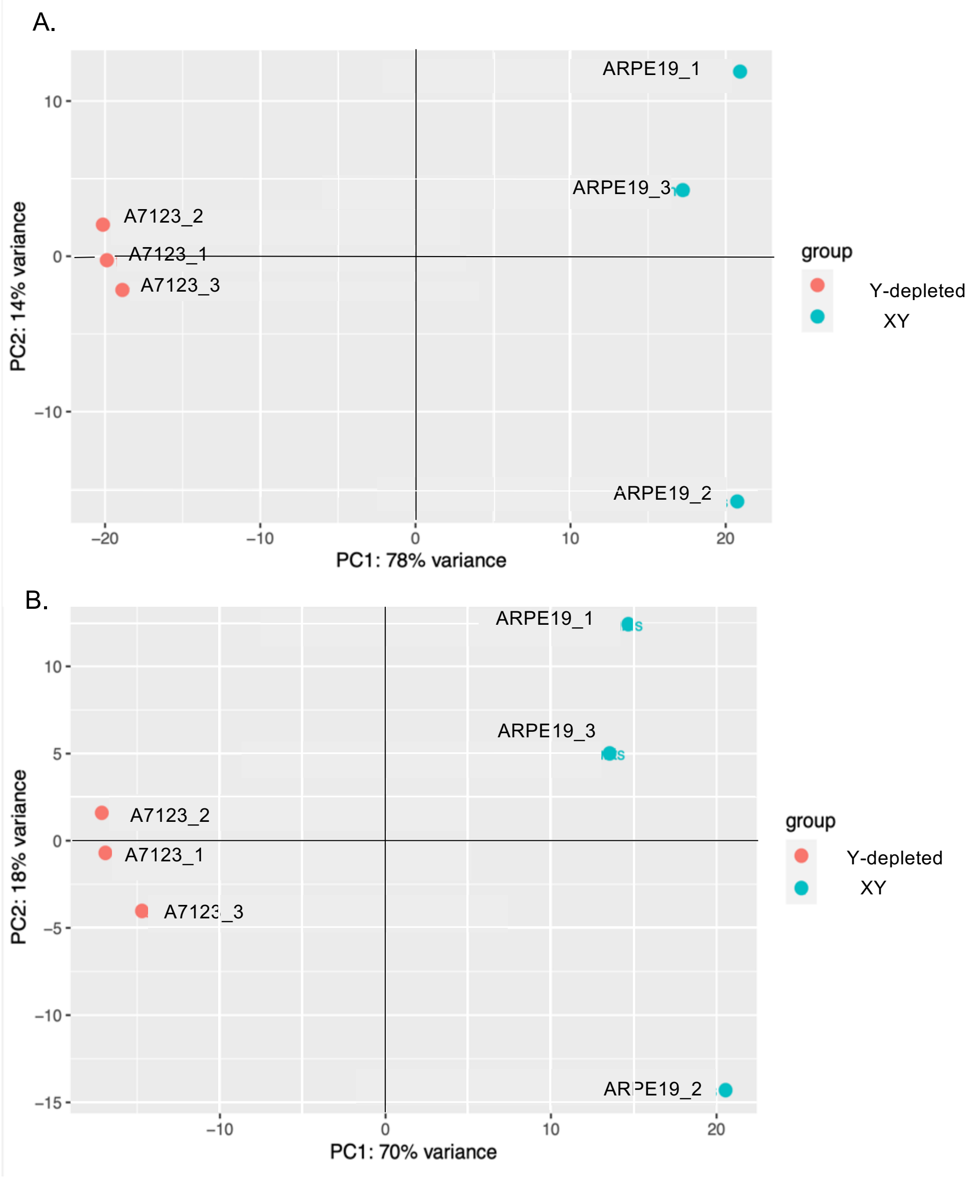

Figure S2. Principal Component Analysis (PCA) graph on gene expression data. A) PCA on PCGs expression data. B) PCA on lncRNAs expression data. In both cases the PC1 explains most of the variance between the samples that are divided according to the condition under investigation.

Figure S3

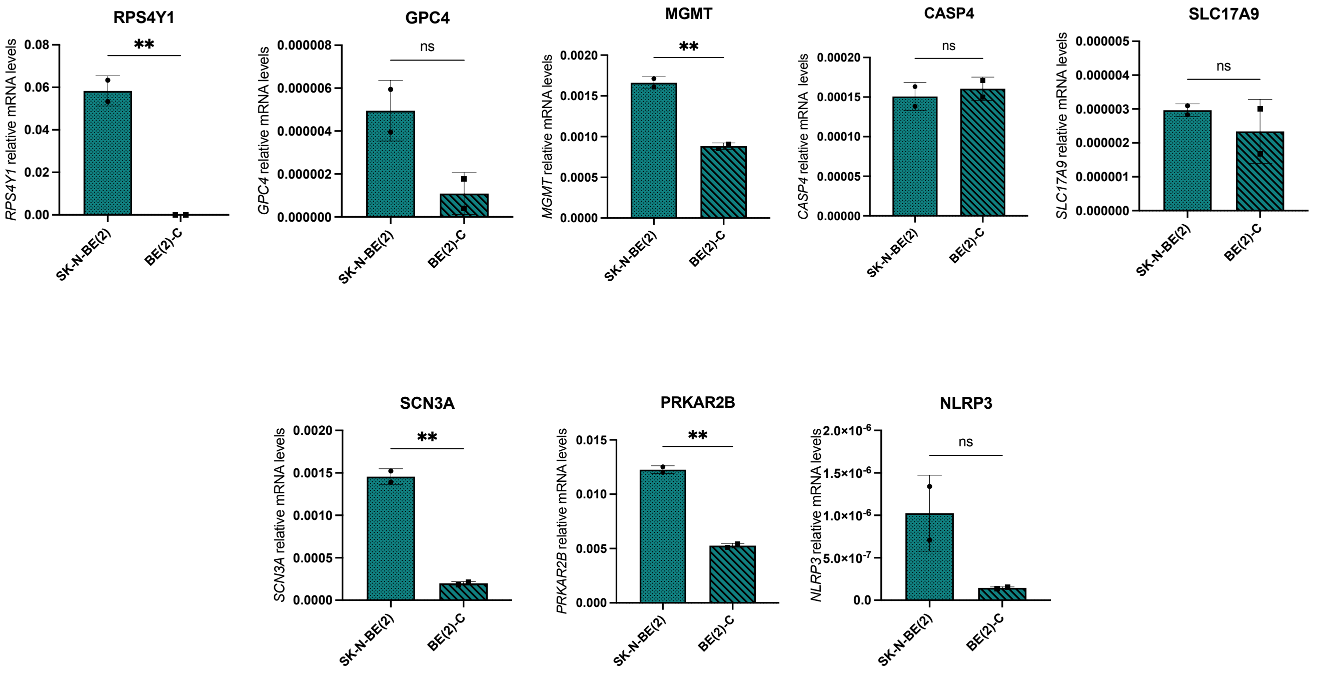

Figure S3. Validation of top deregulated genes in SK-N-BE(2) and Be(2)-C cells. RT-PCR analyses were performed in triplicate by using RNA extracted from Be(2)-C VS SK-N-BE(2). Each bar represents mean ± SD calculated from two independent experiments. P values were derived from Student's t test between the indicated samples (ns: P>0.05; *: P ≤ 0.05; **: P ≤ 0.01; ***: P ≤ 0.001; ****: P ≤ 0.0001).

Figure S4

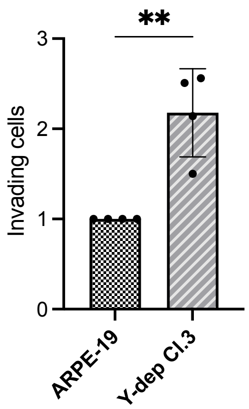

Figure S4. Y chromosome depletion promotes cell invasion. A) Invasion assay followed by crystal violet staining in ARPE-19 and Y-negative clone 3. Cell invasion has been quantified by elution of crystal violet staining with 0,1% SDS and measured at 570 nm absorbance. Each bar represents mean ± SD calculated from four independent experiments. P value was derived from Student's t test between the indicated samples (ns: P>0.05; *: P ≤ 0.05; **: P ≤ 0.01; ***: P ≤ 0.001; ****: P ≤ 0.0001).

Figure S5

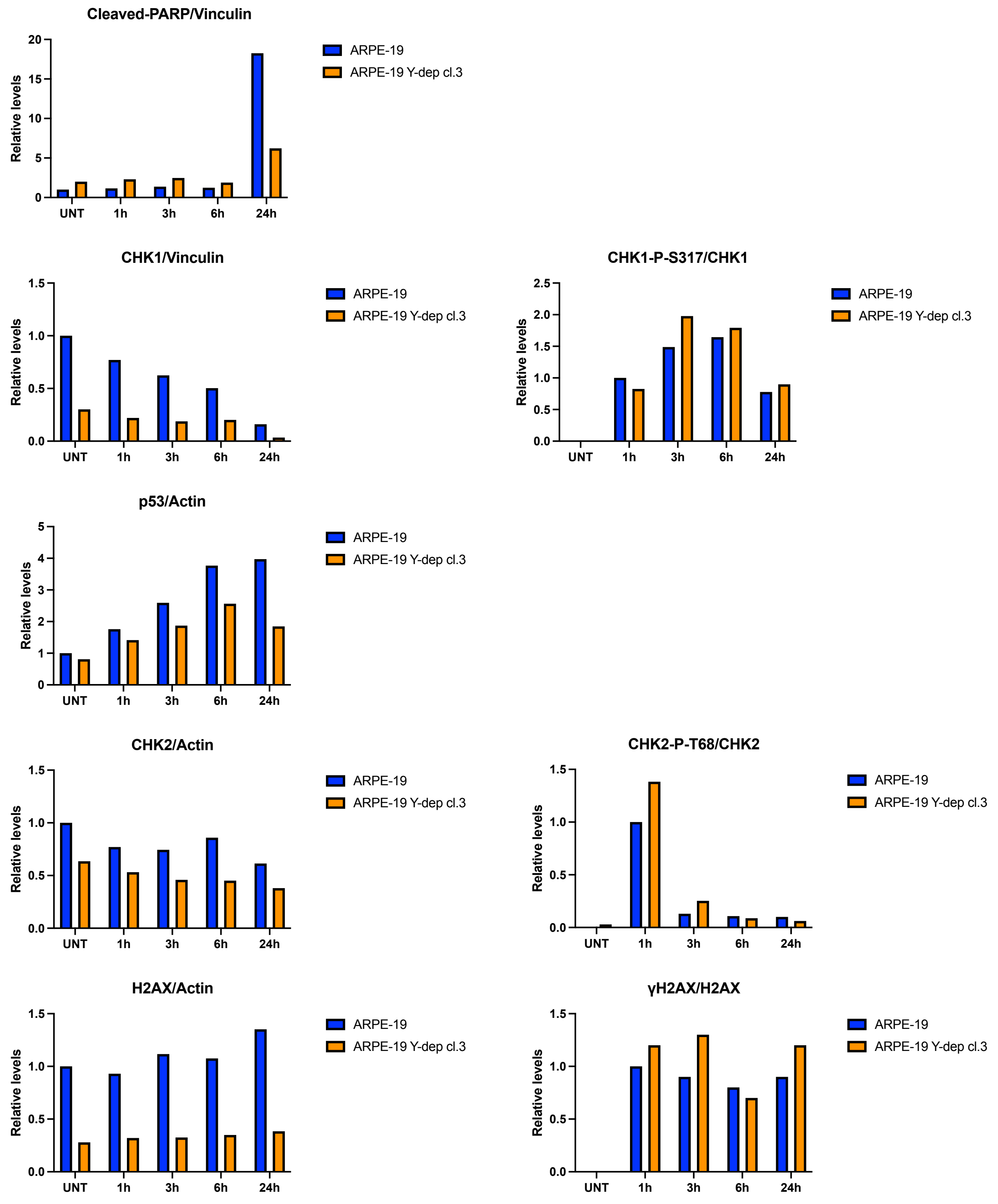

Figure S5. Y chromosome depletion affects DNA damage response to etoposide treatment at different time points. Bar graphs representative of the densitometric analyses of western blots images shown in Fig. 7E. Each graph represents the relative levels of cleaved-PARP, CHK1, p53, CHK2 and H2AX normalized on their respective housekeeping protein, and relative levels of CHK1-P-S317, CHK2-P-T68 and γ-H2AX normalized on their respective total protein.
